# Cosuppression of the chloroplast localized molecular chaperone HSP90.5 impairs plant development and chloroplast biogenesis in Arabidopsis

**DOI:** 10.1186/1756-0500-7-643

**Published:** 2014-09-13

**Authors:** Saehong E Oh, Christine Yeung, Rebecca Babaei-Rad, Rongmin Zhao

**Affiliations:** Department of Biological Sciences, University of Toronto, 1265 Military Trail, Scarborough, Ontario M1C 1A4 Canada

**Keywords:** Molecular chaperone HSP90, Chloroplast biogenesis, Transgene-induced gene silencing, Cosuppression, Transgenic plants, Protein folding, FLAG-tag

## Abstract

**Background:**

HSP90.5 is a chloroplast localized HSP90 family molecular chaperone in Arabidopsis, and it has been implicated in plant abiotic stress resistance, photomorphogenesis and nuclear-encoded protein import into the chloroplast. However, how these processes are controlled by HSP90 is not well understood. To understand the role of HSP90.5 in chloroplast function and biogenesis, in this study, we generated transgenic Arabidopsis plants that overexpress a C-terminally FLAG-tagged HSP90.5. By characterizing three HSP90.5 cosuppression lines, we demonstrated the essential role of HSP90.5 in plant growth and chloroplast biogenesis.

**Results:**

Immunoblotting and quantitative PCR analyses revealed three independent HSP90.5 cosuppressing transgenic lines. All three cosuppression lines displayed a certain degree of variegated phenotype in photosynthetic tissues, and the cosuppression did not affect the expression of cytosolic HSP90 isoforms. HSP90.5 cosuppression was shown to be developmentally regulated and occurred mostly at late developmental stage in adult leaves and inflorescence tissues. HSP90.5 cosuppression also caused significantly reduced rosette leaf growth, transient starch storage, but did not affect rosette leaf initiation or inflorescence production, although the fertility was reduced. Isolation of chloroplasts and size exclusion chromatography analysis indicated that the FLAG at the HSP90.5 C-terminus does not affect its proper chloroplast localization and dimerization. Finally, transmission electron microscopy indicated that chloroplast development in HSP90.5 cosuppression leaves was significantly impaired and the integrity of chloroplast is highly correlated to the expression level of HSP90.5.

**Conclusion:**

We thoroughly characterized three HSP90.5 cosuppression lines, and demonstrated that properly controlled expression of *HSP90.5* is required for plant growth and development in many tissues, and especially essential for chloroplast thylakoid formation. Since the homozygote of HSP90.5 knockout mutant is embryonically lethal, this study provides transgenic lines that mimic the conditional knockout line or siRNA line of the essential HSP90.5 gene in Arabidopsis.

**Electronic supplementary material:**

The online version of this article (doi:10.1186/1756-0500-7-643) contains supplementary material, which is available to authorized users.

## Background

In higher plants, non-photosynthetic proplastids divide and differentiate into specialized plastids in response to developmental and environmental cues [1–3]. Chloroplasts, the photosynthetic plastids, exist in all green tissues and are derived either directly from proplastids in the meristems or from dark-grown intermediates known as etioplasts. In photosynthetic plant cells, chloroplasts also undergo division via a mechanism similar to that used by cyanobacteria, from which the chloroplast originated by symbiosis [4–6]. Chloroplasts facilitate the absorption of solar energy and fix inorganic carbon into organic molecules, thus providing food for all animals.

Different from cyanobacteria genomes which generally encode several thousand proteins, the chloroplast genome contains only 60–200 genes [7]. Chloroplasts in green plant, however, contain several thousand proteins and as many as 95% of them are encoded by nuclear genes and imported from the cytoplasm where they are synthesized [8]. Protein import into the chloroplast is mediated by the Toc/Tic complexes residing in the outer and inner chloroplast membranes [9, 10]. Since proteins are mainly in non-native states during import, refolding is required after translocation. To ensure the proper protein folding and turnover of these proteins as well as newly synthesized ones from its own *de novo* protein synthesis machinery, a comprehensive protein quality control system exists in the chloroplast. This complex system includes chaperone families of HSP40, HSP60, HSP70, HSP90 and HSP100, and proteases ClpP, FtsH, DegP, and SPP [11, 12]. Malfunction of protein quality control components have been shown to impair chloroplast function and plant development [13–15].

HSP90 is a molecular chaperone that has been implicated in playing roles at the late stage of *de novo* protein folding [16]. The HSP90 orthologues have been identified within all known plant chloroplasts and prokaryotic photosynthetic bacteria, and they belong to the HSP90C subfamily [17, 18]. However, since a previous analysis of Arabidopsis genome identified seven HSP90 isoforms, and the chloroplast HSP90 was termed as HSP90.5 [19], we hereafter specifically refer to Arabidopsis chloroplast HSP90 as HSP90.5. HSP90 is composed of three highly conserved domains, an N-terminal ATP-binding domain, a middle domain which has been implicated in binding client proteins, and a C-terminal dimerization domain which helps HSP90 form a homodimer (see reviews by [20–22]). All known eukaryotic cytosolic HSP90s contain a MEEVD motif at the C-terminus that is essential for the binding of proteins with tetratricopeptide repeat (TPR) domains [23, 24], such as cochaperones HOP/Sti1, Cpn6, and Cpn7 which modulate cytosolic HSP90 function [25, 26]. However, this MEEVD pentapeptide motif is lacking in prokaryotic HSP90 isoforms, chloroplast, mitochondrion, and ER-localized HSP90 orthologues. Therefore, the cochaperones that modulate cytosolic HSP90 activity are likely not all conserved in endosymbiont originated organelles.

While it is absolutely required for eukaryotic cells and is generally encoded by a multiple-gene family in higher organisms [19, 27], HSP90 is not essential for prokaryotic cells. HtpG, the prokaryotic HSP90 family member, is absent in many bacteria and *Archaea* [28]. In *E. coli*, HtpG is dispensable and knockout of HtpG only causes slight temperature sensitivity [29]. It is generally believed that the role of HSP90 in prokaryotic cells could be complemented by other chaperones such as the HSP60 family chaperonins.

Nevertheless, HSP90 orthologue in chloroplast is indispensable for eukaryotic photosynthetically active cells. In *C. reinhardii*, a unicellular eukaryote, chloroplast localized HSP90C was identified to form a four-protein ‘foldosome’ [30] with HSP70B, CDJ1 and CGE1 [31]. HSP90C is strongly induced by heat shock, implying a role in stress management [32]. It was proposed that CDJ1 may bring substrate proteins to the HSP90C-HSP70B complex [30], and the functional cycle of HSP90C, if there is one, likely mimics that of cytosolic HSP90, which requires cytosolic HSP70, HOP, p23/Sba1, and other cochaperones [33]. A chlorate-resistant mutant, *cr88*, in Arabidopsis has been shown to result from a point mutation (G646R) in the chloroplast localized HSP90.5 [34]. *cr88* mutant plants have impaired gene expression for photosynthesis associated genes such as nitrate reductase 2 (NR2) and chlorophyll *a/b* binding protein (CAB) [35]. The mutant plants also display long hypocotyls in red light, suggesting its role in the regulation of photomorphogenesis [36]. *HSP90.5* gene has been shown to be constitutively expressed in young plants while its mRNA level is almost undetectable in mature plants [34]. In a recent study by using isolated chloroplasts, HSP90.5 was reported to interact with Tic110, a component of Tic, and cpHSP70, suggesting a role of HSP90.5 in facilitating the import of nuclear encoded proteins into chloroplast and likely in the formation of a foldosome in high plant chloroplast [37].

In an effort to understand the role of HSP90 in plant abiotic stress resistance, we previously generated transgenic Arabidopsis plants that overexpress wild type HSP90.5 and showed that overexpression of HSP90.5 reduces plant tolerance to salt and drought stresses [38]. HSP90.5 overexpressing plants also have impaired resistance to oxidative stress [39]. To further understand the function of HSP90.5 in chloroplast biogenesis and function, in this study, we generated new transgenic Arabidopsis lines that express a C-terminal FLAG-tagged HSP90.5, in which the expression of endogenous and transgenic genes could be differentially monitored. In addition to HSP90.5 overexpression lines, three independent transgenic lines that have their endogenous and transgenic *HSP90.5* expressions cosuppressed at certain development stages were identified. It should be noted that during the revision of this manuscript, Li and colleagues published a work showing that HSP90.5 was also able to be cosuppressed by transforming an untagged HSP90.5 into Arabidopsis, and that HSP90.5 may be involved in the disassembly of VIPP1 [40]. Since analysis of HSP90.5 knockout in Arabidopsis suggested that it is an essential gene and knockout of the gene is embryonically lethal [37], the cosuppression lines obtained in this study mimics a partial loss of function mutant. Our analysis of the cosuppression lines indicated that properly controlled expression of HSP90.5 is important for plant growth and chloroplast biogenesis.

## Results

### Some transgenic plants expressing FLAG-tagged HSP90.5 display variegated phenotype in photosynthetic tissues

**Figure 1:**
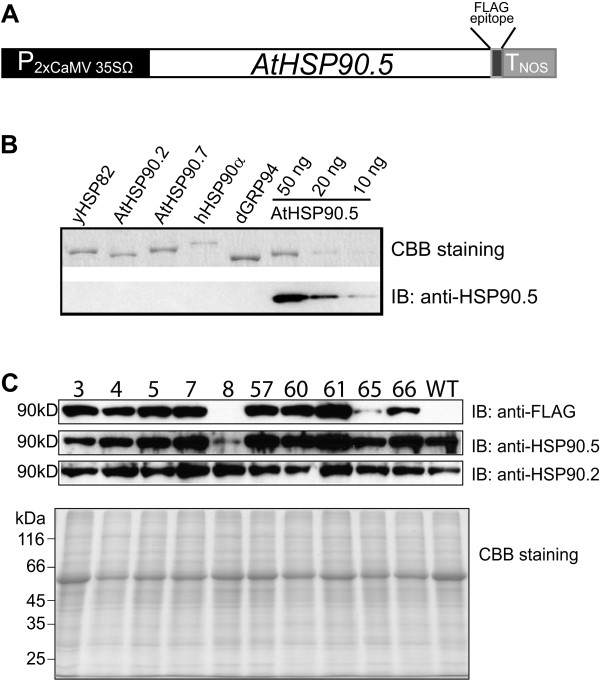
**Screening of primary transgenic plants expressing C-terminally FLAG-tagged HSP90.5. A**, Schematic diagram of C-terminally FLAG-tagged HSP90.5 that was constructed in binary vector pGWB402Ω [41] through gateway cloning system. **B**, Examination of the specificity of polyclonal rabbit anti-HSP90.5 antibody. Immunoblotting was performed using purified cytosolic HSP90 from *S. cerevisiae* (yHSP82), Arabidopsis (AtHSP90.2) and human (hHSP90α), and ER-localized HSP90 from Arabidopsis (AtHSP90.7) and canine (dGRP94), with 50 ng loaded of each, and different amounts of purified AtHSP90.5. CBB staining represents SDS-PAGE stained with Coomassie brilliant blue. **C**, Immunoblotting of total leaf lysate proteins from 3-week-old primary transgenic seedlings. A total of 7.5 μg protein was separated by SDS-PAGE and immunoblotted with anti-FLAG, anti-HSP90.5 and anti-HSP90.2 antibodies.

The initial goal of generating transgenic Arabidopsis plants that overexpress C-terminally FLAG-tagged HSP90.5 is to purify HSP90.5 complex by affinity chromatography and then to determine HSP90.5 interactors via mass spectrometry. To this end, we constructed a binary vector using pGWB402Ω [41] and the final vector contains an *HSP90.5* coding sequence driven by 2xCaMV 35S promoter and a FLAG tag fused at the HSP90.5 C-terminus (Figure 1A). Additionally, in order to investigate how expression of FLAG-tagged HSP90.5 affects the total amount of HSP90.5 *in vivo*, we cloned the *HSP90.5* coding sequence into an *E. coli* expression vector, purified HSP90.5 proteins and raised a polyclonal antibody that specifically recognizes Arabidopsis HSP90.5 (Figure 1B).

**Figure 2:**
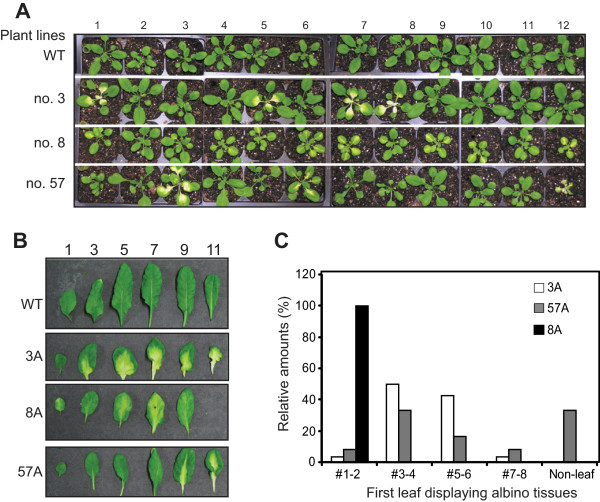
**Appearance of albino tissues in three independent transgenic lines. A**, T2 progenies of transgenic lines no. 3, 8 and 57. 24-day-old seedlings grown under 12/12 hr photoperiod at 110 μmol.m^2^.sec^-1^ and 22°C are shown. **B**, Some rosette leaves (1^st^ vegetative leaf is shown on the left) from wild type (WT) and variegated lines no. 3 (3A), no. 8 (8A) and no. 57 (57A) T3 plants grown for 5 weeks are shown. **C**, Variation of the first vegetative leaf that developed albino tissues in the three transgenic lines. At least 40 plants developing albino tissues from each transgenic line were recorded and analyzed. Non-leaf indicates count for the variegated phenotype that appeared only in inflorescence tissues.

A total of 30 independent kanamycin resistant primary transgenic Arabidopsis plants were screened. Similar to a previous study [38], no obvious phenotype was observed for the primary transgenic plants that were confirmed to contain transgenic FLAG-tagged HSP90.5 gene by PCR genotyping (Additional file 1: Figure S1 and S2A) under normal growth conditions. Immunoblotting using anti-FLAG antibody indicated that FLAG-tagged HSP90.5 was expressed well in most of the transgenic plants (Figure 1C). Interestingly, out of 20 closely examined lines, three transgenic lines, no. 3, 8 and 57, segregated phenotypically in T2 generation into either the wild type like (designated as 3G, 8G or 57G wherever applicable) plants, or those with yellowish, variegated or albino leaves (designated as 3A, 8A or 57A, respectively) (Figure 2A and 2B). Because these plants displayed a conspicuous phenotype and their growth and development were significantly affected, we conducted in-depth analysis on these three transgenic lines in this study.

**Figure 3:**
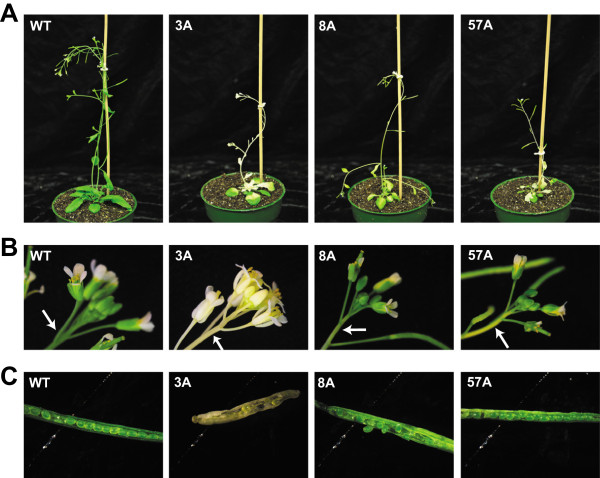
**Variegated transgenic plants at late development stages. A**, Wild type (WT) and variegated lines no. 3 (3A), no. 57 (57A) and no. 8 (8A) plants grown for 37 days under 16/8 hr photoperiod at 110 μmol.m^2^.sec^-1^ and 22°C. **B**, Example albino inflorescence stems and flowers shown for transgenic plants. Arrows indicate the area of albino tissues where they are supposed to be green. **C**, Example siliques from variegated transgenic plants. Lowest (most developed) siliques on primary inflorescence are shown.

Although all three independent lines produced variegated plants in the T2 generation, they developed albino tissues at different developmental stages. At the early developmental stage, the variegated phenotype in line no. 3 appeared in some seedlings when they were 2-week-old, with yellow petiole and albino tissues mostly developing from the base of the 3^rd^ or 4^th^ juvenile leaves. The first few leaves showing albino tissues were still green near the leaf apex and yellow between the albino and green regions (Figure 2B). At a later stage, the emerging adult leaves in line no. 3 were severely serrated and some were completely albino. The albino tissues in line no. 57 usually emerged in some 3-week-old seedlings from the 3^rd^ or 4^th^ vegetative leaf. Unlike line no. 3 plants, however, the albino tissues in the rosette leaves of line no. 57 vary slightly with the yellowish/albino areas generally enriched around the inner part of the leaves along the main vascular tissues (Figure 2B), but also observed to appear on one side from the midvein in some plants. Other plants from line no. 57 that developed the variegation phenotype manifested it in inflorescence tissues at the late developmental stage. Different from lines no. 3 and 57, line no. 8 plants displayed yellowish, mottled phenotype on their vegetative leaves originating from the mid-vein starting from the 1^st^ juvenile leaf (Figure 2B). Interestingly, no yellow/albino cotyledon was observed in any of the three plant lines. In addition to differences between the three lines, variation existed even within the same line. In lines no. 3 and no. 57 plants, the first vegetative leaf to develop albino tissue varied from the 1^st^ to the 7^th^ leaf (Figure 2C), even though plants were maintained under the same growth condition. At the late developmental stage, for all three lines, the albino/yellow tissues also appeared in inflorescence stems, cauline leaves, and sepals (Figure 3). Despite the appearance of albino tissues in many organs, the overall growth and development of those plants showing variegated phenotype were not severely impaired, and most of them were able to flower and produce viable seeds. Only those plants from line no. 3 which had albino tissues initiating from the 1^st^ or the 3^rd^ leaf, a very early onset, were noted to have reduced fertility and difficulty to produce viable seeds compared to other plants, likely due to a deficiency in photosynthetic tissues, resulting in death before seed setting.

To understand how the variegated phenotype is inherited and associated with transgenes, we investigated the segregation of transgenes in the T2 generation by kanamycin resistance test (Table 1) and in the backcrossed lines by PCR genotyping of transgene (example PCR genotyping results are shown in Additional file 1: Figure S2B). It turned out that two variegated lines no. 3 and no. 57 seemed to contain single locus of transgene while no. 8 contained multiple loci of transgenes in the genome. Additionally, variegated phenotype in lines no. 3 and 57 was associated with homozygosity of the transgene, although not all homozygous no. 57 plants show variegated phenotype (Table 1). An in-depth examination indicated that all three transgenic lines displaying albino tissues in the T2 generation were able to produce variegated offsprings up to the 6^th^ generation, which we have analyzed.

**Table 1:** **Segregation of the three** ***HSP90.5*** **cosuppression lines in T2 and T3 generations**

Transgenic lines	Kanamycin sensitivity		Plant growth ^b^		
	Kan ^R^	Kan ^S^	WT-like	Variegated	Variegated plant (%) ^b^
3^a^	85	23	86	27	24
8	82	15	24	113	82
57^a^	172	67	56	12	18
3-12	80	0	0	11	100
3-9	55	0	0	31	100
3-11	77	0	0	20	100
8-4	53	47	3	21	88
8-6	98	2	4	20	83
8-12	77	23	4	20	83
57-3	49	0	7	17	71
57-4	97	0	13	11	46
57-6	63	0	7	17	71

^a^indicates that both segregation ratios of Kan^R^ to Kan^S^ and WT-like to variegated accept the hypothesis of single recessive gene genetics as determined by χ^2^ test. ^b^indicates the plants that were sown and grown directly on soil without kanamycin selection. Lines 3, 8 and 57 represent T2 generation plants and the others represent corresponding T3 generation transgenic plants.

### Variegated phenotype is associated with HSP90.5 cosuppression

**Figure 4:**
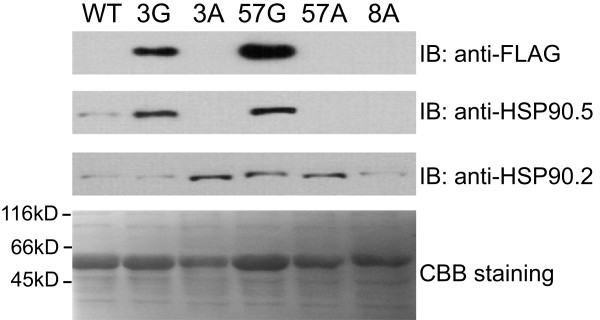
**HSP90.5 expression in mature plants.** Immunoblotting of rosette leaf lysates (6 μg proteins) from variegated (3A, 8A and 57A) and normally growing (3G and 57G) plants of the three transgenic lines using anti-FLAG and anti-HSP90.5, and anti-HSP90.2 antibodies. Total proteins from the 4^th^ vegetative leaves of 4-week-old mature plants were prepared and analyzed. WT represents wild type leaf lysates.

Immunoblotting of primary transgenic plants indicated that the expression level of total HSP90.5 was decreased only in line no. 8, but not in lines no. 3 and 57 (Figure 1C). To understand if the development of albino tissues was associated with altered expression of HSP90.5, we examined the HSP90.5 expression level in rosette leaves from T2 transgenic plants. As shown in Figure 4, the normally growing green leaves from line no. 3 and 57 appeared to express a high level of FLAG-tagged HSP90.5, while the variegated leaves did not show any visible anti-FLAG signal. Interestingly, immunoblotting using anti-HSP90.5 antibody, which is able to detect both endogenous and FLAG-tagged HSP90.5, did not detect any HSP90.5 expression in the albino leaves either. In contrast, immunoblotting using anti-HSP90.2, specific to all cytosolic HSP90.1-4 [38], indicated that the expression of cytosolic HSP90 isoforms was not significantly affected in variegated leaves. This indicated that both endogenous and transgenic *HSP90.5* genes were suppressed in variegated leaves. We hypothesized that the variegated phenotype was induced by a mechanism of transgene-induced cosuppression, which has often been observed in transgenic plant studies [42], and that the cosuppression did not affect the expression of other HSP90 isoforms. It should be noted that anti-HSP90.5 antibody recognizes both endogenous and FLAG-tagged HSP90.5. However, because FLAG-tagged HSP90.5 is only 8-amino-acid longer than the endogenous one, the two isoforms of HSP90.5 in transgenic plants are hardly distinguished by immunoblotting. They were only visible as two close bands occasionally when limited samples were loaded with properly controlled exposure by immunoblotting (data not shown).

**Figure 5:**
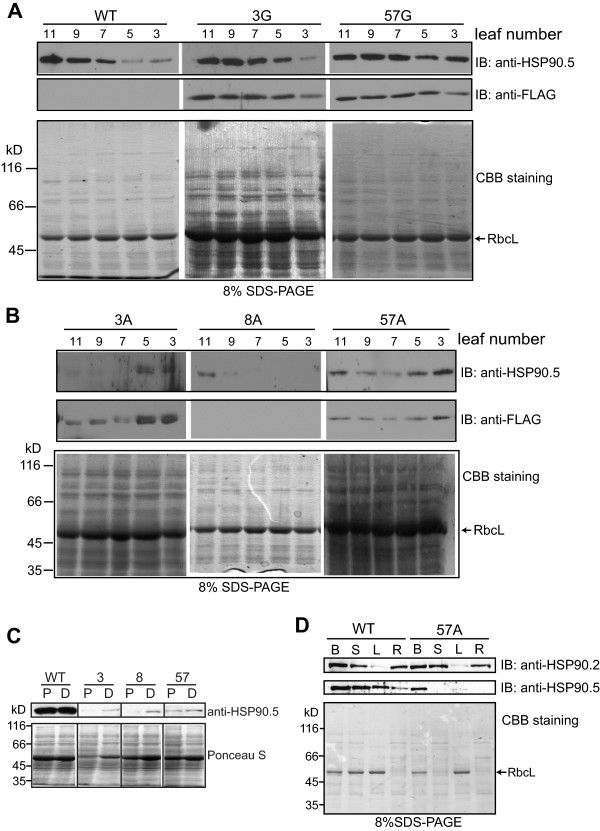
**Tissue specific cosuppression of HSP90.5 in transgenic lines.** Immunoblotting of total soluble proteins (7 μg) from different tissues were analyzed. Anti-FLAG, anti-HSP90.5 or anti-HSP90.2 antibody is used. Plants grown for 3 weeks were analyzed. Wild type (WT), normally growing T3 transgenic lines no. 3 (3G), no. 57 (57G), and variegated transgenic plants no. 3 (3A), no. 57 (57A) and no. 8 (8A) were analyzed. **A** and **B**, Examination of different rosette leaves. **C**, Analysis of different regions of a variegated leaf. The 6^th^ rosette leaf that developed partially albino phenotype was sectioned along the yellowish transition line to separate the albino proximal (P) region from the still green distal (D) region. **D**, Examination of total soluble proteins extracted from unopened flower buds (B), inflorescence stem (S), the 6^th^ leaf (L) and roots (R).

To further understand if cosuppression of *HSP90.5* occurs in all tissues of a variegated plant, we analyzed the HSP90.5 expression in leaves that were initiated at different times. Leaves were numbered from the first vegetative leaf to the 11^th^ leaf from 30-day-old plants grown under a short light cycle. Wild type and normally growing no. 3 and 57 plants showed increasing HSP90.5 expression from the leaves initiated earlier to those initiated later (Figure 5A). This suggests that more HSP90.5 is required for young developing adult leaves, in which chloroplast differentiation and division still actively take place. In contrast, both FLAG-tagged HSP90.5 and total HSP90.5 were decreased in young developing albino adult leaves (7^th^, 9^th^ and 11^th^ leaves) (Figure 5B). For line no. 8 leaves, the HSP90.5 expression level seemed decreased for all leaves. Leaves with decreased HSP90.5 expression appeared to correlate well with the emergence of albino tissues (Figure 2B).

For some juvenile leaves developing albino tissues, the leaf apex remained green (Figure 2, A and B). To examine whether HSP90.5 is differentially suppressed within a leaf, we cut a typical variegated leaf into two regions, the proximal albino/yellow region and the distal green region. HSP90.5 was expressed well in both regions of wild type leaves, however, at a much higher level in the distal region than in the proximal region where albino tissues appeared in cosuppression lines (Figure 5C). This result supports the hypothesis that variegated phenotype is induced by *HSP90.5* cosuppression. Since albino tissues also appeared in other organs such as inflorescence stems and sepals (Figure 3), we examined the expression of HSP90.5 in these tissues. As shown for no. 57 variegated plants, albino flower buds and inflorescence stems had reduced HSP90.5 expression compared to wild type (Figure 5D). Interestingly, HSP90.5 was well expressed in roots of wild type plants, though there is no photosynthesis activity in these tissues, and the expression of HSP90.5 in the roots of variegated plants was noted to be significantly reduced (Figure 5D), suggesting that HSP90.5 cosuppression likely also occurred in root tissues.

**Figure 6:**
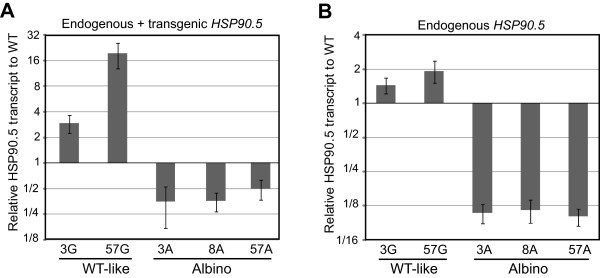
**Relative levels of** ***HSP90.5*** **transcripts in variegated lines.** Relative *HSP90.5* mRNA transcript levels of wild type like no. 3 (3G) and no. 57 (57G) as well as variegated transgenic plants from lines no. 3 (3A), no. 8 (8A) and no. 57 (57A). The transcript levels were normalized to WT transcript level. *ACTIN7* was used as an internal control. Three technical replicates were performed per experiment, and three independent experiments were performed. Error bars represent standard deviation. The graph from a representative experiment is shown. **A**, Total *HSP90.5* mRNA transcript levels. **B**, Endogenous *HSP90.5* mRNA transcript levels.

It should be noted that cosuppression is a phenomenon that usually occurs at the RNA level [43, 44]. To rule out the possibility that the reduced steady state HSP90.5 expression (Figures 4 and 5) was due to abnormal protein degradation, we examined the endogenous and the total *HSP90.5* transcript levels by real time PCR. Total RNAs were isolated from variegated, normally growing transgenic and wild type leaves. As shown in Figure 6A, the variegated leaves from lines no. 3, 8 and 57 all had significantly lower total *HSP90*.5 transcript levels than those from wild type plants, while WT-like transgenic plants (3G, 57G) had significantly higher total *HSP90.5* transcript levels. Additionally, analysis using specific primers designed to detect only the endogenous *HSP90*.5 mRNA transcript (Additional file 1: Table S1 and Additional file 1: Figure S1) showed significantly lower endogenous *HSP90.5* transcript levels in variegated plant lines, compared to WT (Figure 6B), thus providing strong evidence for cosuppression of both endogenous and transgenic *HSP90.5* genes.

### FLAG-tag at HSP90.5 C-terminus does not affect its chloroplast localization and dimerization

**Figure 7:**
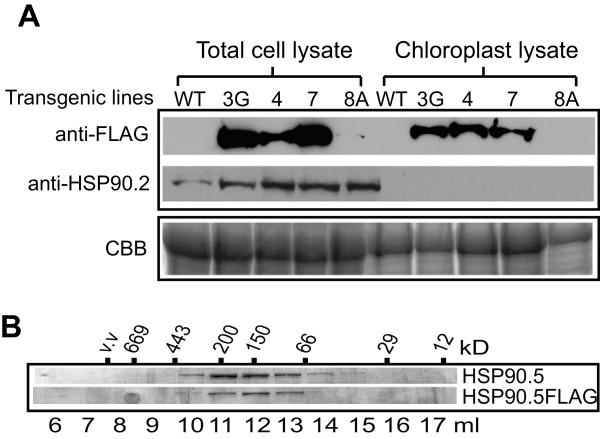
**Chloroplast localization and dimerization of FLAG-tagged HSP90.5. A**, Immunoblotting of total plant leaf lysates and isolated chloroplast lysates from normally growing transgenic lines no. 3 (3G), 4, 7 and variegated no. 8 (8A) lines as well as from wild type (WT) plants using anti-FLAG and anti-HSP90.2 antibodies. SDS-PAGE of 10 μg proteins stained with Coomassie brilliant blue is shown to indicate equal loading. **B**, Size exclusion chromatography of purified mature forms of HSP90.5 and HSP90.5FLAG. The fractions were separated by 12% SDS-PAGE and stained with Coomassie brilliant blue.

To rule out the possibility that variegated phenotype was induced by impaired *in vivo* HSP90 function due to the FLAG-tag at the HSP90.5 C-terminus, we tested for the proper chloroplast targeting and dimerization of FLAG-tagged HSP90.5. We isolated chloroplasts from normally growing 3G heterozygote plants, cosuppression line no. 8 plants, and overexpression lines no. 4 and 7 plants (Figure 1B). FLAG-tagged HSP90.5 was well contained in isolated chloroplasts from all transgenic lines except line no. 8 (Figure 7A). Immunoblotting using anti-HSP90.2, did not detect any signal from isolated chloroplast samples, suggesting that anti-FLAG signal in isolated chloroplast lysates was not due to cytosolic protein contamination. No anti-FLAG signal was detected in line no. 8 chloroplasts. This is reasonable because FLAG-tagged HSP90.5 expression is very low in line no. 8 cosuppression leaves (Figures 4 and 5).

To test whether the C-terminal FLAG-tag affects the dimerization of HSP90.5, we expressed and purified both FLAG-tagged and wild type HSP90.5 from *E. coli*. The purified proteins were subjected to size exclusion chromatography. FLAG-tagged HSP90.5 was eluted with a peak at the molecular mass of about 160kDa, very similar to the elution peak of wild type HSP90.5 (Figure 7B). This indicated that FLAG-tag at the C-terminus did not affect the HSP90.5 dimerization. Additionally, since the C-termini of cytosolic HSP90 in eukaryotes contain extremely conserved MEEVD motifs that are required for the tetratricopeptide repeat domain binding [23, 24], we examined chloroplast HSP90 C-termini that are derived from different species. Alignment of chloroplast HSP90 homologues from green algae, moss, Selaginella, Arabidopsis, rice, maize, soybean, and Populus indicated that the C-terminal amino acids of chloroplast HSP90 are not absolutely conserved (Additional file 1: Figure S4). Combined with its proper chloroplast localization (Figure 7A), and the fact that high level expression of FLAG-tagged HSP90.5 in the heterozygotes of lines no. 3 and no. 57 (Figures 4, 5A) did not affect the plant growth or show variegated phenotype, it is unlikely that the FLAG-tag at C-terminus significantly affects HSP90.5 function *in vivo*. The variegated phenotype in lines no. 3, 8 and 57 is likely only due to reduced protein expression level.

### Cosuppression of HSP90.5 impairs plant growth and chloroplast development

**Figure 8:**
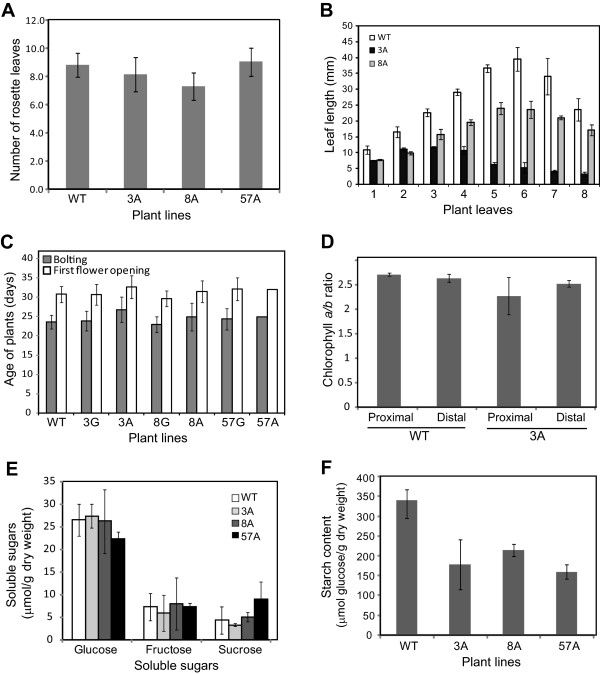
**The growth and development of HSP90.5 cosuppression plants.** Wild type (WT) and variegated transgenic plants from lines no. 3 (3A), no. 8 (8A) and no. 57 (57A) were grown under 16/8 hr photoperiod at 110 μmol.m^2^.sec^-1^ and 22°C. Error bars represent standard deviation. At least 20 plants from each line were analyzed for **A** and **C**. Three independent experiments were performed for B, D, E and F. **A**, The total number of rosette leaves from plants grown for three weeks. **B**, Vegetative leaf length (mm) measured for 40-day-old mature plants. **C**, Ages of plants when they started to bolt and had first flower bud opened. **D**, Chlorophyll *a*/*b* ratios of wild type and variegated line no. 3 rosette leaves. The leaves were sectioned into proximal and distal regions. **E**, Soluble sugar contents in rosette leaves collected at the end of the day. **F**, Insoluble starch contents measured as glucose from the rosette leaves collected at the end of the day.

The overall growth and development of *HSP90.5* cosuppression plants were obviously affected at the late developmental stage (Figure 3), but not significantly affected at the early developmental stage (Figure 2A). To understand how cosuppression of *HSP90.5* affects plant growth, we monitored the total number of rosette leaves that developed in plants showing the variegated phenotype. Interestingly, except that line no. 8 plants developed slightly fewer rosette leaves, variegated plants from lines no. 3 and 57 developed very similar numbers of rosette leaves to WT plants (Figure 8A), although not surprisingly, cosuppression plants had significantly reduced leaf length, particularly for adult rosette leaves (Figure 8B). This suggests that reduced expression of HSP90.5 does not affect rosette leaf initiation. In agreement with this observation, *HSP90.5* cosuppression plants and wild type plants bolted and had their first flower buds opened at almost the same time (Figure 8C). The chlorophyll contents were also measured, and although significantly less chlorophyll *a* and *b* in *HSP90.5* cosuppression leaves were noted compared to those in wild type leaves (Additional file 1: Figure S3), the chlorophyll *a*/*b* ratios in cosuppression leaves were very similar (Figure 8D). Additionally, measurement of soluble sugars and insoluble starch from rosette leaves at the end of photoperiod indicated that, while cosuppression leaves had very similar soluble sugar contents to wild type leaves (Figure 8E), the starch contents in *HSP90.5* cosuppression leaves were significantly reduced (Figure 8F). This suggests that transient starch storage in chloroplasts during the daytime was significantly reduced.

**Figure 9:**
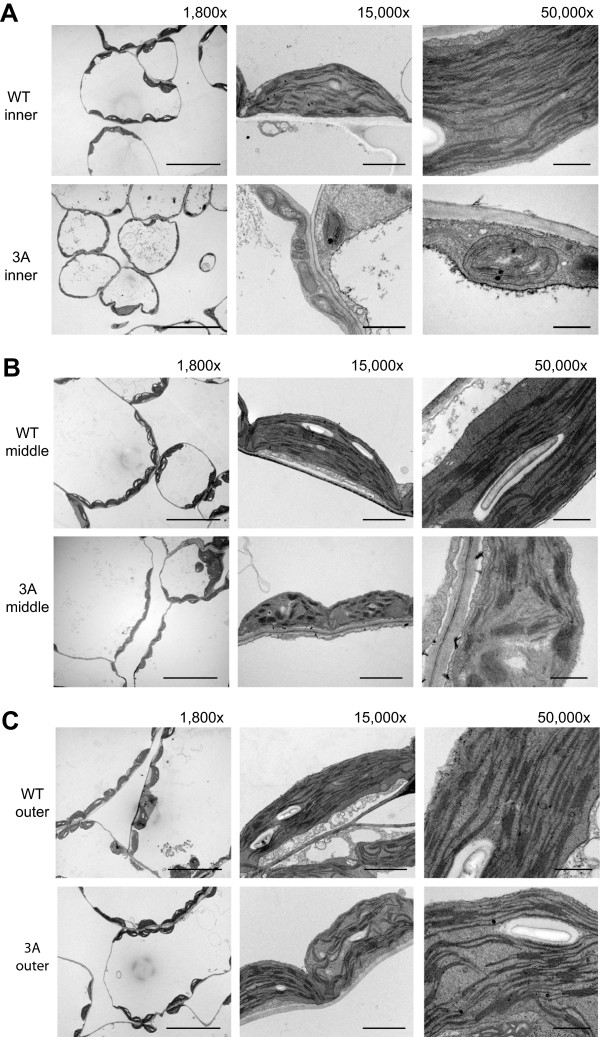
**Transmission electron microscopy of variegated rosette leaves from no. 3 transgenic plants.** The 6^th^ vegetative leaves from 23-day-old variegated transgenic plant line no.3 (3A) and wild type (WT) were sectioned into three regions: inner albino region, middle yellow transition region and outer green region. The scale bars represent 500 nm with 50,000 x, 2 μm in 15,000 x and 20 μm in 1,800 x magnifications. **A**, EM images for inner (proximal) regions. **B**, EM images for middle transition regions. **C**, EM images for outer (distal) green regions.

It has been reported that chloroplasts in the *HSP90.5* point mutation line, *cr88,* are smaller than those in wild type and contain fewer stacked thylakoids in young yellow-green leaves [35]. To determine whether chloroplast development in the albino tissues is defective, thus contributing to impaired transient starch accumulation, we examined the chloroplasts by transmission electron microscopy. We dissected the partially albino 6^th^ rosette leaves into three sections: an inner region (proximal to the petiole) which was the most albino/white, an outer (distal) region which was still green, and a middle transition region, which was yellowish green. Compared to those in wild type, chloroplasts in the inner region mesophyll cells from variegated leaves appeared undeveloped and significantly smaller (~2 μm long in albino leaf while > 5 μm long in wild type) with little thylakoid development and no grana stack formation (Figure 9A). More specifically, the thylakoid membranes of chloroplasts in albino leaf were thinner and only present as single layer fragments (<500 nm long). In the middle transition region of variegated leaves, chloroplasts were larger (~5 μm long) and contained many more thylakoid membranes compared to those in the inner region mesophyll cells. Grana stacks were also recognizable in chloroplasts within the middle transition region, although these stacks appeared disorganized (Figure 9B). In the outer green region of the variegated leaf, the chloroplasts were similar to those of wild type leaves in terms of size and morphology, but the stacks were still not as organized as those in wild type chloroplasts (Figure 9C). In conclusion, the albino region of the *HSP90.5* cosuppression leaves had a reduced number of properly developed chloroplasts in mesophyll cells and *HSP90.5* cosuppression seemed to be tightly associated with the impaired chloroplast development.

## Discussion

### Mis-regulation of molecular chaperone HSP90 expression in Arabidopsis leads to pleitropic effects

In this study, we identified and analyzed three *HSP90.5* cosuppression lines and showed that reduced expression of HSP90.5 impairs chloroplast development (Figure 9) and plant growth (Figure 8B), and induces an abnormal variegated phenotype in some rosette leaves and inflorescence tissues where they are supposed to be green and photosynthetically active (Figures 2A and 3). Because of abnormal chloroplast development and function, the transient starch contents in *HSP90.5* cosuppression leaves are also significantly reduced (Figure 8, E and F). This study therefore establishes the essential role of the HSP90 family protein in chloroplast biogenesis, and development. It should be noted that the variegated phenotype of *HSP90.5* cosuppression lines is different from the delayed greening phenotype observed for the *cr88* mutant. The typical *cr88* mutant phenotype is the slower greening process than wild type throughout the life span. For *HSP90.5* cosuppression plants, the albino tissues mainly appear in the late developmental stage and never develop in the cotyledons, while *cr88* seedlings have yellowish cotyledons [35]. In agreement with this phenotype, we did not observe any significant repression of HSP90.5 expression in cotyledons of the variegated lines by immunoblotting (data not shown). More importantly, whenever the variegated phenotype appears in leaves, stems or sepals, it remains and does not become green at the later time.

By immunoblotting analysis, we showed that the appearance of albino tissues is highly correlated to reduced HSP90.5 protein levels (Figure 5). However, this does not indicate that HSP90.5 is not required for non-photosynthetic tissues. Since HSP90.5 is well expressed in root and flower tissues (Figure 5D) of wild type plants, this suggests that molecular chaperone HSP90 may also play a role in the function of other types of plastids such as amyloplasts and chromoplasts. Our results agree with the microarray analysis of *HSP90.5* transcript [45] and imply that plastid localized molecular chaperone HSP90 may play a global role for plastid division, differentiation and physiological function, as has been observed for the global roles of cytosolic HSP90 in fungi [46] and mammalian cells [47, 48]. Based on a recent study on HSP90.5 in protein import into chloroplast [37], and our observation that HSP90.5 is also cosuppressed in the root tissues of line no. 57A plants (Figure 5D), it would be interesting to examine whether and how the development of the other type plastids such as amyloplast in HSP90.5 cosuppressing plants is affected.

### Role of HSP90.5 in thylakoid formation and retrograde signaling pathway

Thylakoid membrane formation within the plastid is a hallmark of chloroplast differentiation. The biogenesis of the thylakoid network is a complex process that is poorly understood. While the insertion of photosynthetic proteins into existing thylakoids has been noted [49], little is known about the formation of the thylakoid membrane itself. Nonetheless, we now know that thylakoid biogenesis requires coordination of protein synthesis, translocation of lipid and pigment synthesis [50, 51]. In Arabidopsis, VIPP1, a vesicle-inducing plastid protein, has been shown to play important roles in thylakoid formation [52]. A *vipp1* mutation in Arabidopsis also displays variegated phenotypes with very few internal membranes or only distorted/degraded thylakoids. VIPP1 does not display any features of an intrinsic membrane protein, but is associated with chloroplast membranes, suggesting its role in transport of lipids from the inner membrane to the thylakoids [53]. Studies in *C. reinhardti* have shown that chloroplast HSP90 forms a complex with HSP70 and VIPP1, implicating HSP90.5 in thylakoid membrane formation [54]. Electron microscopy analysis in our study showed that *HSP90.5* cosuppression albino regions in rosette leaves lack thylakoid membranes, and that only partially developed thylakoid membranes exist in the yellowish-green transition region (Figure 9), thus in agreement with previous studies on roles of HSP90.5 and VIPP1 in thylakoid membrane formation [35, 52].

The *cr88* mutant developed longer hypocotyls in red light, but not in far-red or blue light, suggesting that *HSP90.5* plays a role in photomorphogenesis [35], likely by affecting phytochrome PhyB signaling but not PhyA signaling [55]. By analyzing *cr88cop1* double mutant, it was demonstrated that HSP90.5 likely functions downstream of COP1 [36], a master regulator in photomorphogenesis [56]. Impaired expression of photosynthesis-associated genes in the *cr88* mutant [35] supports the role of HSP90.5 in photomorphogenesis. However, it is intriguing that HSP90.5 is localized in plastids while all studied photomorphogenesis-related proteins such as COP1, DET1 and photoreceptors [56, 57] are localized in the cytoplasm and nucleus. Whether the observed effect of *cr88* on photomorphogeneis results from an indirect consequence of impaired chloroplast development, or whether *cr88* plants directly exerts a retrograde signal to regulate photomorphogenesis-related gene expression is unclear. The role of HtpG, a molecular chaperone HSP90 orthologue, in regulating the activity of uroporphyrinogen decarboxylase and thus regulating tetrapyrrole biosynthesis [58] has been noted in cyanobacteria [59]. A potential retrograde signal, the chlorophyll biosynthesis intermediate Mg-protoporphyrin IX has been shown to bind cytosolic HSP90 [60], chloroplast HSP90.5, and even HSP70 [61] in Arabidopsis. Therefore, it is very likely that reduction of HSP90.5 expression in cosuppression lines directly impairs the retrograde signaling pathway mediated by Mg-protoporphyrin IX. The three *HSP90.5* cosuppression lines with distinct variegated phenotype would allow deciphering the role of HSP90 in chlorophyll biosynthesis and in the chloroplast retrograde signaling pathway in the future, in addition to its known role in nuclear encoded protein import into the chloroplast [37].

## Conclusions

In this study, we showed that the plastid-localized HSP90.5 is widely expressed in Arabidopsis plants, and transgene-induced silencing of HSP90.5 significantly impairs plant growth and chloroplast biogenesis in the leaves. By analyzing the differential silencing levels of HSP90.5, we demonstrated that properly controlled expression of *HSP90.5* is required for plant growth and development. This study provided further evidence showing the essential role of HSP90.5, and generated transgenic lines that mimic the conditional knockout line of the essential HSP90.5 gene in Arabidopsis, thus allowing us to further investigate the function of HSP90.5 in the future.

## Methods

### FLAG-tagged HSP90.5 gene construction and plant materials

C-terminally FLAG-tagged HSP90.5 coding sequence was amplified by PCR from an *HSP90.5* cDNA clone [38] with primers HSP90.5GWFullF and HSP90.5GWFullR (Additional file 1: Table S1). The FLAG-tag (DYKDDDDK) coding sequence was in primer HSP90.5GWFullR and the amplified fragment was cloned using gateway cloning strategy and finally cloned into destination vector pGWB402Ω [41], generating pGWB402Ω-HSP90.5FLAG binary vector, with *HSP90.5* controlled by 2xCaMV 35S promoter and NPTII gene for screening of transgenic plants. *Arabidopsis thaliana* ecotype Columbia-0 (*Col-0*) was transformed with *A. tumefaciens* GV3101 carrying pGWB402Ω-HSP90.5FLAG using the floral dip method [62]. All plants were grown within Conviron ATC60 growth chamber under 16/8 hr photoperiod at 110 μmol.m^2^.sec^-1^ and 22°C unless specified. To select transgenic plants or test kanamycin resistance, seeds were sterilized, sown onto ½ Murashige and Skoog salt (MS salt, Sigma) agar medium with 1% sucrose and 25 μg/ml kanamycin and grown under 120 μmol.m^2^.sec^-1^ at 16/8 hr photoperiod and 22°C in Adaptis 1000 growth incubator.

### Protein expression and purification from *E. coli*and antibodies

The coding region of predicted mature HSP90.5 (D62-D780) was amplified from the cDNA construct and cloned into p11 vector [63] and that of FLAG-tagged mature form was amplified from pGWB402Ω-HSP90.5FLAG and cloned into pProEXHTb, generating expression plasmids p11-HSP90.5 and pProEXHTb-HSP90.5^62-780^FLAG, respectively. Primers used for cloning and the relative positions of primers are shown in Additional file 1: Table S1 and Additional file 1: Figure S1. N-terminally His_6_-tagged mature forms of HSP90.5 and HSP90.5FLAG were expressed in and purified from *E. coli* using Ni-NTA (QIAGEN) according to the manufacturer’s manual. The purified proteins were used either for size exclusion chromatography or for polyclonal antibody production by Signalway AntiBody (College Park, USA). Other primary antibodies used in this study include anti-FLAG monoclonal antibody (Sigma), and anti-HSP90.2 antibody, which was originally raised using purified *S. cerevisiae* HSP82 [46], but also specifically recognized Arabidopsis cytosolic HSP90.1 and HSP90.2, while not HSP90.5 [38].

### Chloroplast isolation and quantification of chlorophyll

The protocol for chloroplast isolation from Arabidopsis rosette leaves was adapted from Kley et al. [64]. Briefly, approximately 10 g of 2-week-old Arabidopsis seedlings were collected and crushed in 1X Clp buffer (330 mM sorbitol, 50 mM HEPES/KOH, pH 7.5, 5 mM EDTA, 5 mM EGTA, 1 mM MgCl_2_, 10 mM NaHCO_3_, and 0.5 mM DTT). The homogenate was filtered through two layers of Miracloth (Calbiochem) before it was loaded on the top of 40% Percoll™ layer (made with 1X Clp buffer) and centrifuged at 2,000 × g for 10 minutes. The chloroplasts in the pellets were washed with 10 mL 1X Clp buffer and centrifuged at 1,000 × g for 5 min. The pellet was frozen in liquid nitrogen and stored at -80°C until further use.

To measure chlorophyll contents from whole leaf lysate, the leaves were homogenized in liquid N_2_ and then suspended in 80% acetone. The solution was centrifuged for 5 minutes at 15,000 g at 4°C. The supernatant was collected and absorptions at 663 nm and 646 nm were measured using Nanodrop 2000c (Thermo Scientific). The total chlorophyll (*a* + *b*) level, the chlorophyll *a* and chlorophyll *b* contents were estimated according to previously described [65].

### Total soluble protein extraction from fresh leaves and immunoblotting analysis

Leaf or inflorescence tissues were ground in an eppendorf tube using a micropestle with 5× fresh weight of buffer containing 100 mM Tris–HCl, pH7.5, 20 mM EDTA, 100 mM NaCl, 0.1% β-mecaptoethanol, 1× protease inhibitor cocktail (Bioshop). The mixtures were centrifuged for 10 min at 14,000 rpm and the supernatants were collected. Protein concentrations were determined by Bradford reagents (Bio-Rad) and samples were subjected to SDS-PAGE, transferred to nitrocellulose membrane and immunoblotted with specific antibodies.

### Size exclusion chromatography

The protein samples were run on a size exclusion column, Superdex™ 200 10/30 (GE Healthcare), with the assistance of ÄKTApurifier 10 (GE Healthcare). The column was pre-equilibrated with running buffer (25 mM Tris–HCl, pH 8.0, 100 mM NaCl, 10% glycerol) and 600 μg of HSP90.5^62–780^ or 200 μg of C-terminally FLAG-tagged HSP90.5^62–780^ was run. Fractions in 0.5 ml were collected and analyzed by SDS-PAGE. Protein standards used are as previously described [66].

### Complementary DNA synthesis and Real-Time PCR analysis

Total RNA was extracted with RNeasy® Mini Kit (QIAGEN) combined with QIAshredder (QIAGEN) from approximately 100 mg (fresh weight) of leaf samples that were frozen in liquid N_2_ and ground to a fine powder using a micropestle. Superscript first-strand synthesis system (Invitrogen) was used for the synthesis of cDNA from 250 ng of total RNA. cDNA (6.3 ng) was added to iQ SYBRE Green qPCR supermix (Bio-Rad). Forward primer HSP90.5-qF was used in combination with reverse primer HSP90.5-qEndo-R to detect endogenous mRNA, or reverse primer HSP90.5-qTotal-R to detect the total mRNA. The primer sequences and their relative positions in *HSP90.5* gene are indicated in Additional file 1: Table S1 and Additional file 1: Figure S1. The mean C_t_ (cycle threshold) value of three replicates was normalized against the C_t_ for *ACTIN7*, an internal control (Additional file 1: Table S1). Data analysis was performed with Bio-Rad Gene Expression Analysis for iCycler iQ® Real-Time PCR Detection System (version 1.10).

### Hexose, sucrose and starch analyses

To measure the soluble sugar and transient starch contents in Arabidopsis, fresh rosette leaves were collected at the end of the light cycle and snap frozen in liquid nitrogen followed by lyophilisation for 5 hr. The soluble hexose, sucrose and starch in the rosette leaves were measured following the procedure described by Wang et al. [67].

### Transmission electron microscopy (TEM)

Leaves from 23-day-old plants were dissected and prepared for transmission electron microscopy analysis at the Centre for Neurobiology and Stress (CNS), University of Toronto Scarborough (UTSC). TEM sample preparation was performed according to Hyman & Jarvis [68]. Hitachi H7500 Transmission Electron Microscope was used with Olympus SIS Megaview II digital camera. The samples were visualized on iTEM software.

## Electronic supplementary material

Additional file 1: **One supplemental file is provided including the following supplemental table and figures:** **Table S1.** Sequence of primers used for cloning, transgene detection and quantitative reverse transcription-PCR. **Figure S1.** The Arabidopsis HSP90.5 gene structure with relative positions of primers used for this study. **Figure S2.** PCR amplification of transgene fragments from the genomic DNA to genotype transgenic plants. **Figure S3.** Chlorophyll a and b contents in rosette leaves of HSP90.5 cosuppression lines. **Figure S4.** Sequence alignment of chloroplast HSP90 homologues from different species. (PDF 4 MB)
