# Supplementary material for: Cosuppression of the chloroplast localized molecular chaperone HSP90.5 impairs plant development and chloroplast biogenesis in Arabidopsis
Source: BMC Res Notes. 2014 Sep 13;7:643. doi: 10.1186/1756-0500-7-643 (PMC4168064; doi:10.1186/1756-0500-7-643)
Supplement: Supplementary file 1 — Additional file 1: One supplemental file is provided including the following supplemental table and figures: Table S1. Sequence of primers used for cloning, transgene detection and quantitative reverse transcription-PCR. Figure S1. The Arabidopsis HSP90.5 gene structure with relative positions of primers used for this study. Figure S2. PCR amplification of transgene fragments from the genomic DNA to genotype transgenic plants. Figure S3. Chlorophyll a and b contents in rosette leaves of HSP90.5 cosuppression lines. Figure S4. Sequence alignment of chloroplast HSP90 homologues from different species. (PDF 4 MB) [file 13104_2014_3160_MOESM1_ESM.pdf]

Table S1 Primers used in this work

Name	Sequence (5' to 3')	Purpose
HSP90.5GWFullF	GGGGACAAGTTTGTACAAAAAAGC AGGCTCCATGGCTCCTGCTTTGAGTAGAAGT	Used to amplify the AtHSP90.5 full length cDNA for BP reaction to generate entry vector. The underlined represent the FLAG-tag coding sequence.
HSP90.5GWFullR	GGGGACCACTTTGTACAAGAAAGCTGGGTTTACTTGTCATC ATCGTCCTTATAGTCATCTTGCCAAGGATCACTCTC	
HSP90.5K478F	CAAGAAATCTCTGAGAGTGAA	Transgene detection
HSP90.5K625R	TTGCAACTTTGTCACCGAGC	
HSP90.5D62F	GCAACCATGGACGCCGCCGTGGCGGAG	Clone C-terminally FLAG-tagged and non-tagged mature form (D62-D780) into pProEXHTb vector
HSP90.5D780FLAGR	CCGTTCTAGATTACTTGTCATCATCGTCCTT	
HSP90.5D780R	CCGTTCTAGATCAATCTTGCCAAGGATCACTCTC	
HSP90.5-qF	GTGGATTCACTCCTGATAGC	Forward primer for qPCR
HSP90.5-qEndo-R	CACTGCAAACAAAAGAAGG	Reverse primer for endogenous gene (3’ UTR) for qPCR
HSP90.5-qTotal-R	GCCAAGGATCACTCTCTGCC	Reverse primer for both endogenous and transgene for qPCR
ACTIN7F	TCACAGAGGCACCTCTTAACC	Reference gene for qPCR
ACTIN7R	CCCTCGTAGATTGGCACAG	

Figure S1

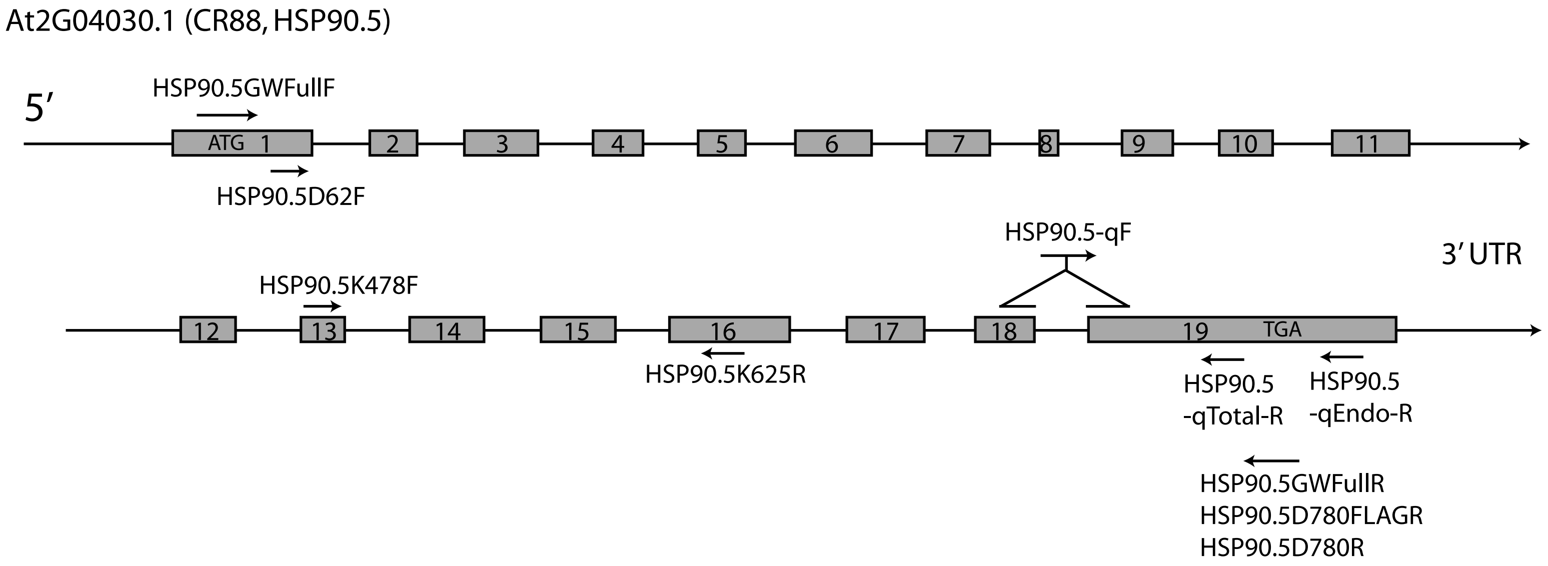

Figure S1. The Arabidopsis HSP90.5 gene structure with relative positions of primers used for this study. The numbered boxes represent exons. The relative positions of translation initiation codon ATG and stop codon TGA are also labelled.

Figure S2

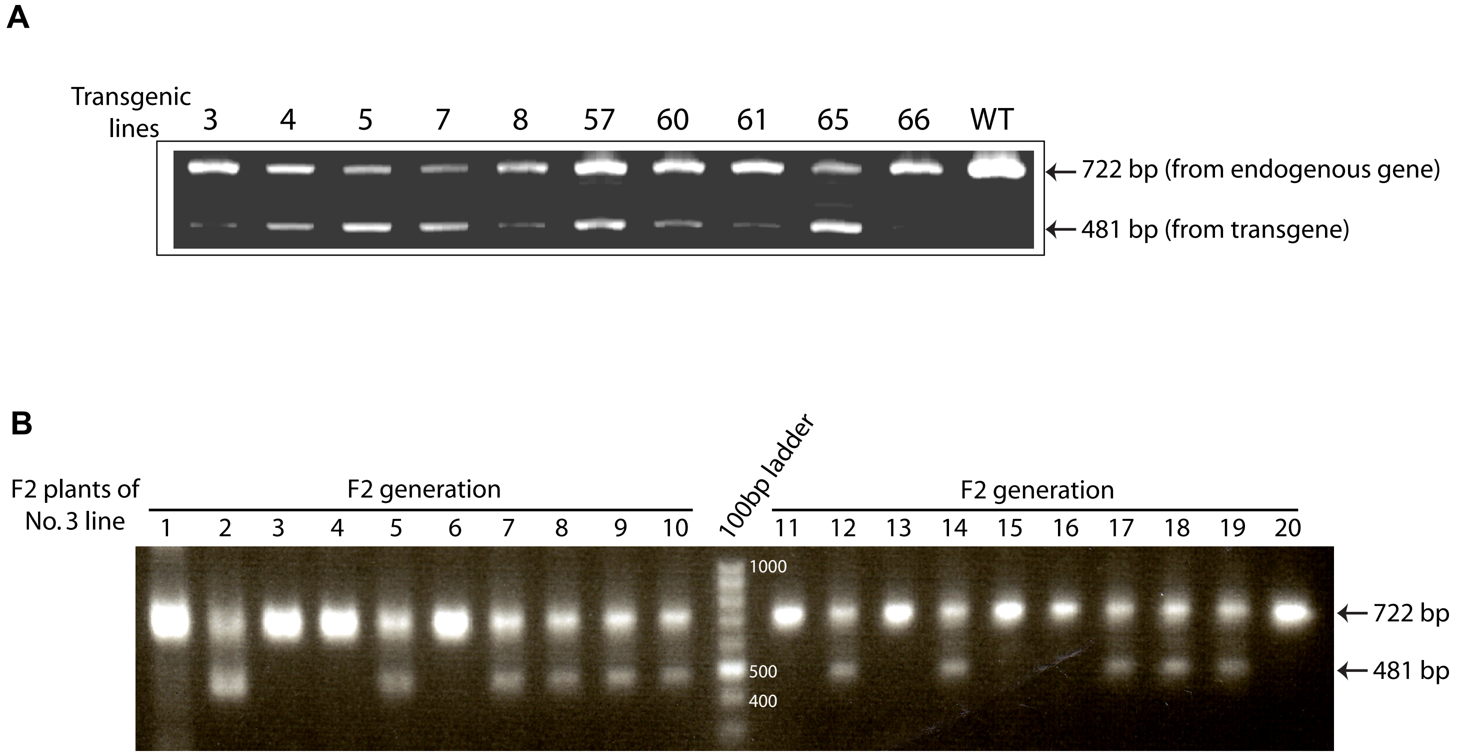

Figure S2. PCR amplification of transgene fragments from Arabidopsis genomic DNA to genotype transgenic plants. A, A typical PCR analysis for primary transgenic plants. B, A typical PCR analysis of the F2 generation plants from backcrossed heterozygous transgenic lines. PCR amplification using HSP90.5K478F and HSP90.5K625R produced a 722bp fragment from endogenous HSP90.5 gene and a 481bp fragment from transgene.

Figure S3

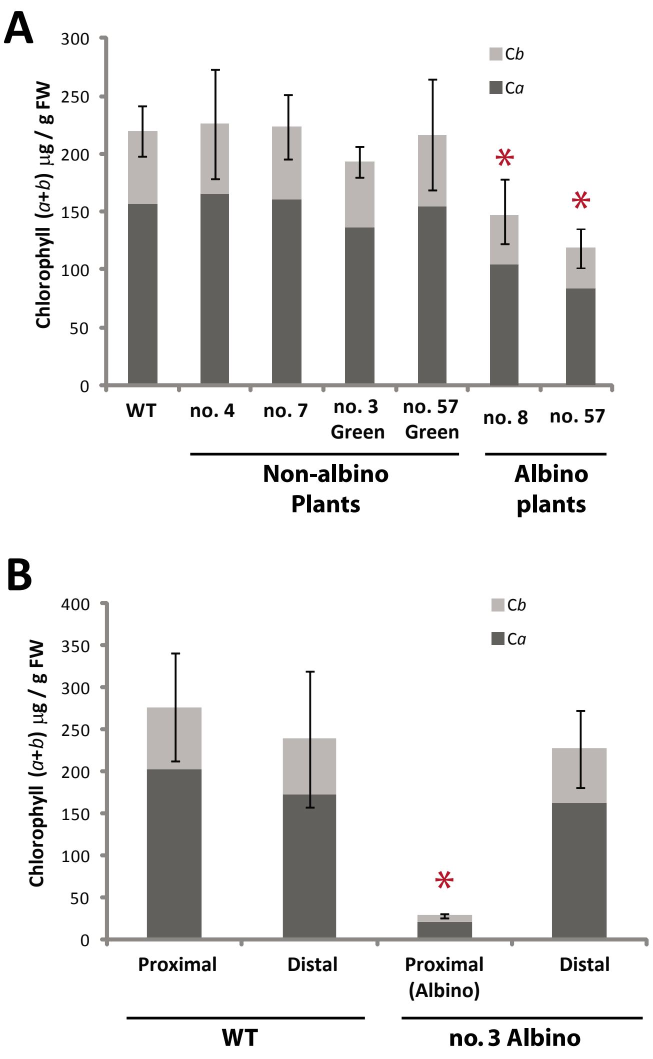

Figure S3. Chlorophyll a and b contents in rosette leaves of HSP90.5 cosuppression lines. 5-week-old plants were grown at 110 µmol.m^2.sec^{-1} with 16/8 hr light/dark cycle.Error bars represent standard deviation with three independent assays. *denotes significantly different ($P < 0.05$) compared to WT values by student t-test. C*a*: Chlorophyll *a*. C*b*: Chlorophyll *b*.

A, Entire rosette leaves of transgenic plants were analyzed for chlorophyll content.

B, For Wild type (WT) and no. 3 alino plants, the leaves were sectioned into proximal (albino) and distal portions for chlorophyll extraction to differentiate the albinism.

Figure S4.

```
AtHSP90.5   611 LLCDWIKQQLGDKVAKVQVSNRLSSSPCVLVSGKFGWSANMERLMKAQALGDTSSLEFMRGRRILEINPD 680
PtHSP90C    619 LLCDWIKQQLGEKVAKVQVSKRLSSSPCVLVSGKFGWSANMERLMKAQALGDQSSLEFMRGRRILEINPD 688
GmHSP90C    620 LLCDWIKQQLGDKVAKVQISNRLSSSPCVLVSGKFGWSANMERLMKAQALGDTASLEFMRGRRILEINAD 689
OsHSP90C    611 LLCDWVKQQLGDKVAKVQISKRLSLSPCVLVSGKFGWSANMERLMKAQTLGDTSSLEFMRGRRIFEINPD 680
ZmHSP90C    618 LLCDWVKQQLGDKVAKVQISKRLSSSPCVLVSGKFGWSANMERLMKAQTLGDTSSLEFMRGRRIFEINPD 687
PpHSP90C    664 LLCDWMKQNLGDKVAKVTVSKRISSSPCVLVSGKFGWSANMERIMKAQTLGDNSQMEFMRGRRILEINPN 733
SmHSP90C    640 YCCDWIKQILGEKVASVGISNRLSTSPCVLVTGKHGWSANMERIMKAQALGDTSQLDYMRGKRILEINPQ 709
CrHSP90C    629 PVVDFLKKALGERVEKVTVSNRLLDSPCALVTSKFGWSANMERIMRSQALGDARAMEYMKGRKIMEINPN 698
                *::*: **::* .* :*:*:  ***.**:.*.********:*::*:***   :::*:*::*:***.:*.:

AtHSP90.5   681 HPIIKDLNAACKNAPESTEATRVVDLLYDTAIISSGFTPDSPAELGNKIYEMMAMAVGGRWGRVEEEEES 750
PtHSP90C    689 HPIIKDLNAACKNAPDSSDAKRAVDLLYDTALISSGFTPDSPAELGGKIYEMMAMALGGRWGRSDGDEAE 758
GmHSP90C    690 HPIIKDLSAACKNAPDSSEAKRAVDLLYDTALISSGFSPDSPAELGNKIYEMMALALGGRWGRSEEEEGD 759
OsHSP90C    681 HPIVKDLNAACKNEPESTEAKRAVELLYETALISSGYTPDSPAELGGKIYEMMTIALGGRWGRSDTETEA 750
ZmHSP90C    688 HPIIKDLSAACKNEPESTEARRAVELLYEAALISSGYTPESPAELGGKIYEMMAIALGGRWGRSDMEEAE 757
PpHSP90C    734 HPIIQDLNVACKDTPNNPRAQAMVNLLHETALLSSGFTPENPAEFGARVYEMMGLALIGKQGGEEKAESV 803
SmHSP90C    710 HPIIASLNEACKSSPHDTRAQEIVELLYETAHVSSGFTPDNASEFGARIYDMIGVALGGRQVLSGQEEEY 779
CrHSP90C    699 HDIIAGIKTLLKEKDED-RARDLSELLYETALITSGFQVDSPKDYASKVFTLMKIALGYDILSEAEEQAA 767
                * *: .:.   *.  ..  *    :**:::* ::**:  :.. : . ::: :: :*:

AtHSP90.5   751 STVNEGDDKSG----------------------------ETEVVEPSEVRA---ESDPWQD-- 780
PtHSP90C    759 DNAEESDANAS-------------------------ETSEPQVIEPSEVRT---ESDPWQD-- 791
GmHSP90C    760 ASVEAADSSTSE------------------------ESSEPEVYEPSEVIA---ESDPWTTD- 794
OsHSP90C    751 ATTGDASTETGS-----------------------SEATVTEVIEPSEVRP---ESDPWRD-- 785
ZmHSP90C    758 ASTGEASAEADS-----------------------SEGTVAEVIEPSEVRP---ESDPWRDQ- 793
PpHSP90C    804 EQSTPSEEGATS-------------------------EVPSEVVEASEVIA---ERDPWQS-- 836
SmHSP90C    780 SAPSAPQVDYSQGYSGGYGVSPPPPEASAATPPPPPATEAEVVVEPSEVR----EGDPWKS-- 836
CrHSP90C    768 AAAPQAAEAAAAP--------------------KAAEAAAVPKVEATPVDAEVVSDDPWKKSA 810
                                                            *.: *     . ***
```

Figure S4. Sequence alignment of the C-terminal fragments of chloroplast HSP90C isoforms from different species.

The alignment was performed using CLUSTALW (http://npsa-pbil.ibcp.fr/cgi-bin/align_clustalw.pl) and only partial C-terminal fragments are shown. The HSP90C isoforms are as following with accession numbers included in the brackets: AtHSP90.5, *Arabidopsis thaliana* HSP90.5 (Q9SIF2); PtHSP90C, *Populus trichocarpa* HSP90C (XP_002311417); GmHSP90C, *Glycine max* HSP90C (XP_0035188021); OsHSP90C, *Oryza sativa* HSP90C (B8BC47); ZmHSP90C, *Zea mays* HSP90C (AFW651291); PpHSP90C, *Physcomitrella patens* HSP90C (XP_0017798941); SmHSP90C, *Selaginella moellendorffii* HSP90C (D8TAK0); CrHSP90C: *Chlamydomonas reinhardtii* HSP90C (Q66T67). The relative positions of the amino acids are also labelled for each isoforms. "*" represents identical amino acids. "." and ":" represent weak and strong similarity respectively.
